# Prevalence data of diarrheagenic *E. coli* in the fecal pellets of wild rodents using culture methods and PCR assay

**DOI:** 10.1016/j.dib.2020.106439

**Published:** 2020-10-22

**Authors:** Md Mafizur Rahman, Sang Jin Lim, Wook Han Kim, Jae Youl Cho, Yung Chul Park

**Affiliations:** a Division of Forest Science, Kangwon National University, Chuncheon 24341, Republic of Korea; b National Institute of Crop Science, Rural Development Administration, Jeonju, Republic of Korea; c Department of Genetic Engineering, Sungkyunkwan University, Chunchun-Dong 300, Suwon 440-746, Republic of Korea; d Faculty of Biological Scienec, Dept. of Biotechnology and Genetic Engineering, Islamic University, Kushtia 7003, Bangladesh

**Keywords:** Wildlife, *Apodemus agrarius*, *E. coli*, Shiga toxin-producing *E. coli*, Virulence genes

## Abstract

Wild animals, such as rodents seem to be competent reservoir of bacteria-borne zoonotic diseases which disseminate in human. We investigated the presence of *E. coli*, Shiga toxin-producing *E. coli* (STEC), and *Salmonella* in the feces of six category wild rodent species (*Apodemus agrarius, A. peninsulae, A. sylvaticus, Micromys minutus, Myodes regulus,* and *R. norvegicus*) captured from different agricultural regions in South Korea. Among them, *A. agrarius*, which account for 65% of total (*N* = 52) individuals, are most widely distributed and abundant in various agroecosystems in South Korea. The bacterial identification was performed by cultural and molecular methods. In cultural method, the fecal cultures from 26 individuals formed colonies on *E. coli*-selective EMB agar media. Of them, the fecal cultures from 18 individuals also produced colonies on the Shiga toxin-producing *E. coli*-selective CT-SMAC agar media as well as the EMB agar media. In molecular method, polymerase chain reaction (PCR) was carried out to detect two virulence genes (*stx1* and *stx2*) of isolated *E. coli*. The amplified dataset of *stx1* and *stx2* genes of *E. coli* were sequenced. In this manuscript, *E. coli* and STEC were detected but there were no *Salmonella* species. The wild rodents’ data would provide important information on reservoirs of those pathogenic bacteria.

## Specifications Table

**Table:** 

Subject area	Microbiology
Specific subject area	Molecular Microbiology, Diarrheagenic *E. coli*
Type of data	Table, Word, and Figure
How data were acquired	Culture methods, PCR assay, and Sequencing
Data format	Raw and Analyzed
Parameters for data collection	Morphological identification, gene specific PCR amplification and screening, sequencing the target isolates, and the sequences were compared with other homologous sequences deposited in GenBank using BLASTN2.2.31+ [1]
Description of data collection	We captured wild rodents using Sherman trap in various agricultural and mountainous area in South Korea. The *E. coli* and Shiga toxin-producing *E. coli* (STEC) bacteria in the fecal samples were detected by culturing them using a method described in a previous study [2]. The universal marker of bacterial 16S rRNA genes was amplified with HVR (V1SF and V3AR) primer set for molecular identification. Only a single pure colony of all 26 positive *E. coli* samples was used for PCR amplification. Shiga toxin genes with Stx1 and Stx2 primer sets were used for detection of STEC isolates. One or more pure colonies were randomly selected from all of positive individuals.
Data source location	Sobaeksan national park (36°56′45.2′' N, 128°27′43.9′'E) in Yeongju, Gyeongsangbuk-do; Gayasan national park (35°48′00.4′' N, 128°08′30.5′'E) in Geochang, Gyeongsangnam-do; Odesun national park (37°47′31.79′' N, 128°32′20.99′'E) in Pyeongchang, Gangwon-do, South Korea.
Data accessibility	Data are with this article only. Repository Name: NCBI The hypervariable region (HVR) of 16 s RNA gene (V1SF and V3AR) sequences were deposited to under the accession number: KY885178 to KY885185 (direct link from https://www.ncbi.nlm.nih.gov/nuccore/KY885178 to https://www.ncbi.nlm.nih.gov/nuccore/KY885185) and the accession number: KY048442 to KY048446 (direct link from https://www.ncbi.nlm.nih.gov/nuccore/KY048442 to https://www.ncbi.nlm.nih.gov/nuccore/KY048446). The Shiga toxin-producing genes (*stx1*) were deposited to under the accession number: KY964457 to KY964469 (direct link from https://www.ncbi.nlm.nih.gov/nuccore/KY964457 to https://www.ncbi.nlm.nih.gov/nuccore/KY964469) The Shiga toxin-producing genes (*stx2*) sequences were deposited to under the accession number: MN266867 to MN266871 (direct link from https://www.ncbi.nlm.nih.gov/nuccore/MN266867 to https://www.ncbi.nlm.nih.gov/nuccore/MN266871) Anyone can see the supplementary data and sequence fasta file to direct URL: Repository Name: [Mendeley Data] Data identification number: DOI: 10.17632/9dkfkfnyzs.2 Direct URL to data: http://dx.doi.org/10.17632/9dkfkfnyzs.2
Related research article	M.M. Rahman, K.B. Yoon, S.J. Lim, M.G Jeon, H.J. Kim, H.Y. Kim, Y.C. Park, Molecular detection by analysis of the 16S rRNA gene of fecal coliform bacteria from the two Korean Apodemus species (*Apodemus agrarius* and *A. peninsulae*). Genet. Mol. Res. 16 (2) (2017), gmr16029510. https://doi.org/10.4238/gmr16029510.

## Value of the Data

• The data will provide important information on competent reservoir of several bacteria-borne zoonotic diseases in wild rodent species and their fecal might play an important role in the transmission of pathogens, such as diarrheagenic *E. coli* bacteria.
• The dataset provides information about prevalence of diarrheagenic *E. coli* and could be used for better management of microbial contamination control.
• The data will contribute to understanding the potential factors that lead to increase in food-borne illness of farmers during agricultural production and processing.

## 1 Data Description

Wild rodents are competent reservoirs of zoonotic diseases that are responsible for significant economic losses and public health problems [3]. The rodents, such as *Apodemus agrarius* and *A. peninsulae*, are very common in South Korea and are widely distributed across agricultural farm areas and mountainous forests [4]. They can disseminate zoonotic microorganisms that are a considerable threat to the health of farmers [3,4]. Therefore, they could be important potential factors that lead to increases in food-borne illness during agricultural production and processing. However, wild rodents of *A. agrarius*, which account for 34 (65%) of the 52 captured individuals, were most commonly captured in the fields (Table 1 and 5). The *E. coli* and Shiga toxin-producing *E. coli*, bacteria in the rodent fecal samples were detected by culture and molecular method. For cultural identification, the *E. coli* or Shiga toxin-producing *E. coli* colonies were confirmed based on their colony morphology. The *E. coli* colonies produced metallic sheen color produced on EMB agar media. Sorbitol negative colonies (colorless) were detected onto CT-SMAC agar media (Supplement Fig. S1). We select colorless colony as *E. coli* O157:H7 positive colonies seem to be STEC-positive on culture media. Moreover, the bacterial colonies on the selective media that had been identified by their morphology were re-identified by PCR using molecular markers. So, a PCR amplification of the bacterial 16S rRNA gene was performed to confirm whether the colonies on the EMB media belonged to *E. coli*. We randomly selected a single colony from each of the 26 positive EMB agar plates (Table 1) and after PCR amplification with the hypervariable region (HVR) of 16S primer set, the target bands for the 16S rRNA gene were found in all 26 single colonies (Supplement Fig. S2). Thirteen PCR bands were then sequenced (Table 2). A PCR amplification of the Shiga toxin genes (*stx1* and *stx2*) was performed to confirm whether the colonies on the CT-SMAC media belonged to Shiga toxin-producing *E. coli*. The target PCR bands of 21 *E. coli* with *stx1* gene and 5 *E. coli* with *stx2* gene were amplified and sequenced (Supplement Fig. S3 and S4). Of them, 13 Stx1 PCR bands and 5 Stx2 PCR bands were sequenced (Table 3 and 4). The *E. coli* and Shiga toxin-producing *E. coli* sequences were compared for similarity with bacteria deposited in GenBank using NCBI BLAST, which is available at http://www.ncbi.nlm.nih.gov/.

**Table 1:** Culture and molecular detection *E. coli*, and Shigh toxin-producing *E. coli* in the rodent fecal samples collected from different environments.

						Culture detection		Molecular detection		
						*E. coli*	Shiga toxin *E. coli*	*E. coli*	Shiga toxin producing *E. coli*	
Individual No.	Species	Fecal sample ID	Sex	Agricultural environment	Collection locality	EMB	CT-SMAC	16S rRNA gene	Shiga toxin gene (*stx1*)	Shiga toxin gene (*stx2*)
1	*Apodemus agrarius*	MuApAg-1	Male	Dry area	Odaesun	×	×	–	–	–
2	*A.agrarius*	MuApAg-2	Male	Nearby forest	Odaesun	×	×	–	–	–
3	*A.agrarius*	MuApAg-3	Male	Nearby forest	Odaesun	×	×	–	–	–
4	*A.agrarius*	MuApAg-4	Female	Dry area	Odaesun	×	×	–	–	–
5	*A.agrarius*	MuApAg-5	Male	Dry area	Odaesun	×	×	–	–	–
6	*A.agrarius*	MuApAg-6	Female	Watery area	Odaesun	×	×	–	–	–
7	*A.agrarius*	MuApAg-7	Male	Nearby forest	Gayasan	o	o	o	×	×
8	*A.agrarius*	MuApAg-8	Male	Nearby forest	Gayasan	×	×	–	–	–
9	*A.agrarius*	MuApAg-9	Male	Nearby forest	Gayasan	×	×	–	–	–
10	*A.agrarius*	MuApAg-10	Male	Dry area	Gayasan	o	×	o	×	×
11	*A.agrarius*	MuApAg-11	Female	Dry area	Gayasan	o	o	o	×	×
12	*A.agrarius*	MuApAg-12	Male	Dry area	Gayasan	o	o	o	×	×
13	*A.agrarius*	MuApAg-13	Male	Watery area	Gayasan	o	o	o	×	o
14	*A.agrarius*	MuApAg-14	Male	Nearby forest	Bukhansan	o	o	o	×	×
15	*A.agrarius*	MuApAg-15	Male	Nearby forest	Bukhansan	o	o	o	×	×
16	*A.agrarius*	MuApAg-16	Male	Nearby forest	Bukhansan	o	o	o	×	×
17	*A.agrarius*	MuApAg-17	Male	Nearby forest	Bukhansan	o	o	o	×	×
18	*A.agrarius*	MuApAg-18	Male	Nearby forest	Sobaeksan	o	o	o	×	×
19	*A.agrarius*	MuApAg-19	Male	Nearby forest	Sobaeksan	o	o	o	×	×
20	*A.agrarius*	MuApAg-20	Male	Nearby forest	Sobaeksan	o	×	o	×	×
21	*A.agrarius*	MuApAg-21	Male	Nearby forest	Sobaeksan	×	×	–	–	–
22	*A.agrarius*	MuApAg-22	Male	Nearby forest	Sobaeksan	o	×	o	×	×
23	*A.agrarius*	MuApAg-23	Male	Watery area	Sobaeksan	o	o	o	×	×
24	*A.agrarius*	MuApAg-24	Female	Nearby forest	Sobaeksan	o	×	o	×	×
25	*A.agrarius*	MuApAg-25	Male	Nearby forest	Sobaeksan	o	×	o	×	×
26	*A.agrarius*	MuApAg-26	Male	Nearby forest	Sobaeksan	o	×	o	×	×
27	*A.agrarius*	MuApAg-27	Female	Nearby forest	Sobaeksan	o	×	o	×	×
28	*A.agrarius*	MuApAg-28	Female	Nearby forest	Sobaeksan	o	o	o	×	×
29	*A.agrarius*	MuApAg-29	Male	Nearby forest	Sobaeksan	×	×	–	–	–
30	*A.agrarius*	MuApAg-30	Male	Nearby forest	Sobaeksan	×	×	–	–	–
31	*A.agrarius*	MuApAg-31	Male	Nearby forest	Sobaeksan	×	×	–	–	–
32	*A.agrarius*	MuApAg-32	Female	Nearby forest	Sobaeksan	×	×	–	–	–
33	*A.agrarius*	MuApAg-33	Female	Nearby forest	Sobaeksan	×	×	–	–	–
34	*A.agrarius*	MuApAg-34	Male	Nearby forest	Sobaeksan	×	×	–	–	–
35	*A.peninsulae*	MuApPe-1	Male	Nearby forest	Odaesan	o	×	o	×	×
36	*A.peninsulae*	MuApPe-2	Female	Dry area	Odaesan	×	×	–	–	–
37	*A.peninsulae*	MuApPe-3	Male	Nearby forest	Sobaeksan	×	×	–	–	–
38	*A.peninsulae*	MuApPe-4	Male	Dry area	Sobaeksan	×	×	–	–	–
39	*A.peninsulae*	MuApPe-5	Male	Nearby forest	Sobaeksan	×	×	–	–	–
40	*A.peninsulae*	MuApPe-6	Female	Watery area	Sobaeksan	×	×	–	–	–
41	*A. sylvaticus*	MuApSy-1	Male	Nearby forest	Gayasan	o	o	o	×	×
42	*A. sylvaticus*	MuApSy-2	Male	Dry area	Gayasan	o	o	o	×	×
43	*A. sylvaticus*	MuApSy-3	Female	Watery area	Gayasan	o	o	o	×	o
44	*Micromys minutus*	MuMiMi-1	Male	Dry area	Gayasan	×	×	–	–	–
45	*M. minutus*	MuMiMi-2	Male	Nearby forest	Gayasan	×	×	–	–	–
46	*M. minutus*	MuMiMi-3	Male	Watery area	Gayasan	×	×	–	–	–
47	*Myodes regulus*	CrMyRe-1	Male	Nearby forest	Bukhansan	×	×	–	–	–
48	*M. regulus*	CrMyRe-2	Male	Nearby forest	Bukhansan	×	×	–	–	–
49	*M. regulus*	CrMyRe-3	Feamle	Nearby forest	Bukhansan	o	o	o	×	–
50	*Rattus norvegicus*	MuRaNo-1	Male	Watery area	Gayasan	o	o	o	×	×
51	*R. norvegicus*	MuRaNo-2	Female	Nearby forest	Gayasan	×	×	–	–	–
52	*R. norvegicus*	MuRaNo-3	Male	Dry area	Gayasan	o	o	o	×	×
**Total individual (N)**						**26**	**18**	**26**	**0**	**2**

'o'= detected, ' × '= not detected and '-'= not tested.

**Table 2:** Molecular identification of *E. coli* in the rodent fecal samples using 16 s rRNA gene sequences.


									Similarity analysis in GenBank			

Species	No. of host individuals used for fecal culture	No. of host individuals with fecal *E. coli-*positive colonies on EMB	No. of host individuals with *E. coli*-positive PCR band	No. of the sequences obtained from PCR bands	No &Host individual ID	No& Sequenced colony ID	Size (bp)	GenBank accession No.	Coverage (%)	Identity (%)	Identification of bacteria by the similarity analysis of 16 s rRNA gene sequences in GenBank	Remarks
*Apodemus agrarius*	34	19	19	7	MuApAg-7	M1–6	539	KY048445	100	100	*E. coli* MRY15–131 (AP017620.1)	General *E. coli*
					MuApAg-18	M_1_Sobeksan-edit	584	KY885178	100	100	*E. coli* AR_0104 (CP020116.1)	
					MuApAg-19	M_2_S	569	KY885179	100	100	*Escherichia* sp. BAB-6436 (KY672923.1)	
					MuApAg-20	M_3_Sobeksan_edit	604	KY885180	100	100	*E. coli* AR_0104 (CP020116.1)	
					MuApAg-22	M_4_S	611	KY885181	100	100	*E. coli* NGF1(CP016007.1)	
					MuApAg-23	M_6_S	539	KY048444	100	100	*E. coli* MRY15–131 (AP017620.1)	
					MuApAg-27	M_10_s	585	KY885182	100	100	*E. coli* 20Ec-P-124 (AP017610.1)	
*A. peninsulae*	6	1	1	1	MuApPe-1	M2M-3	579	KY048443	100	99	*E. coli* NGF1 (CP016007.1)	
*A. sylvaticus*	3	3	3	2	MuApSy-1	MWM-1	454	KY048442	100	100	*E. coli* BLR(DE3) (NZ_CP020368.1)	
					MuApSy-2	MWM2-iii	600	KY885185	100	100	*E. coli* MDR_56 (CP019903.1)	
*Micromys minutus*	3	×	–	–	–	–	–	–	–	–	–	
*Myodes regulus*	3	1	1	1	CrMyRe-3	SFM	565	KY048446	100	99	*E. coli* BLR (DE3) (NZ_CP020368.1)	
*R. norvegicus*	3	2	2	2	NuRaNo-1	SFM_3_BKNP	571	KY885183	100	100	*E. coli* 20Ec-P-124 (AP017610.1)	
					NuRaNo-3	SFM_4_BKNP	498	KY885184	100	99	*E. coli* sch21(JX294878.1)	
**Total (N)**	**52**	**26**	**26**	**13**	**11**	**13**	–	–	–	–	–	–

' × '= not detected and '-'= not tested.

**Table 3:** Molecular identification of Shiga toxin-producing *E. coli* in the rodent fecal samples using Shiga toxin gene (*stx1*) sequences.


									Similarity analysis in GenBank				

Species	No. of the host individuals used for fecal culture	No. of host individuals with fecal Shiga toxin *E. coli-*positive colonies on CT-SMAC	No. of host individuals with the colonies with Shiga toxin gene (*stx1*)-amplified PCR bands	No. of the sequences obtained from the Shiga toxin gene (*stx1*)- amplified PCR bands	Host individual ID	Sequenced colony ID	GenBank accession No.	Size (bp)	Coverage (%)	Identity (%)	Identified genes and their products were found in GenBank	Identification of bacteria by the similarity analysis of Shiga toxin gene (*stx1*) sequence in GenBank	Remarks
*Apodemus agrarius*	34	12	12	5	MuApAg-7	SFM1_2	KY964461	570	100	99	*p^bpc^* (penicillin-binding protein 1C)	*E. coli* strain AH01 (CP055251.10)	Pathogenic *E. coli*
					MuApAg-11	SFMd2–1	KY964458	302	100	100	*allE* (*S*)-ureidoglycine aminohydrolase	*E. coli* strain MS6192 (CP054940.1)	
					MuApAg-12	SFMd3–24	KY964459	342	100	100	*p^bpc^* (penicillin-binding protein 1C)	E. coli strain MS6192 (CP054940.1)	
					MuApAg-13	SFMW-16	KY964460	364	100	99	*p^bpc^* (penicillin-binding protein 1C)	*E. coli* strain AH01 (CP055251.10	
					MuApAg-18	M1_S	KY964457	267	100	99	*pphc* gene (protein‑serine/threonine phosphatase PphC	*E. coli* strain 04–00,955 (CP035498.1)	
*A. peninsulae*	6	0	–	–	–	–	–	–	–	–			
*A. sylvaticus*	3	3	3	5	MuApSy-1	MWM1_10	KY964462	504	100	99	*p^bpc^* (penicillin-binding protein 1C)	*E. coli* strain 88–3510 (CP027675.1)	
					MuApSy-2	MWM2_4	KY964463	223	100	98	*pphc* gene (protein‑serine/threonine phosphatase PphC	*E. coli* strain SCU-484(CP051744.1)	
					MuApSy-3	MWM2_5	KY964464	311	100	99	Regulator gene	*E. coli* strain EcPF7(CP054232.1)	
						MWM3_7	KY964465	581	100	99	p^bpc^ (penicillin-binding protein 1C)	*E. coli* strain SCU-316 (CP054371.1)	
						MWM3_9	KY964466	584	100	99	p^bpc^ (penicillin-binding protein 1C)	*E. coli* strain SCU-316(CP054371.1)	
*Micromys minutus*	3	0	–	–	–	**–**	**–**	**–**	**–**	**–**			
*Myodes regulus*	3	1	1	0	CrMyRe-3	**×**	**–**	**–**	**–**	**–**			
*R. norvegicus*	3	2	2	3	MuRaNo-1	NRW1–14	KY964467	420	100	99	*allE* (*S*)-ureidoglycine aminohydrolase	*E. coli* strain MS6192(CP054940.10	
						NRW1_LB	KY964468	248	100	100	*pphc* gene (protein‑serine/threonine phosphatase PphC	*E. coli* strain SCU-316 (CP054371.1)	
					MuRaNo-3	NRW3–15	KY964469	274	99	99	*pphc gene* (protein‑serine/threonine phosphatase PphC	*E. coli* strain SCU-316(CP054371.1)	
Total (N)	52	18	18	13		–	–	–	–	–	–	–	–

' × '= not detected and '-'= not tested.

**Table 4:** Molecular identification of Shiga toxin-producing *E. coli* in the rodent fecal samples using Shiga toxin gene (stx2) sequences.


									Similarity analysis in GenBank				

Species	No. of the host individuals used for fecal culture	No. of host individuals with fecal Shiga toxin producing *E. coli-*positive colonies on CT-SMAC	No. of host individuals with the colonies with Shiga toxin gene (*stx2*)- amplified PCR bands	No. of the sequences obtained from the Shiga toxin gene(*stx2*)- amplified PCR bands	Host individual ID	Sequenced colony ID	GenBank accession No.	Size (bp)	Coverage (%)	Identity (%)	Identified genes and their products were found in GenBank	Identification of bacteria by the similarity analysis of Shiga toxin gene (*stx2*) sequence in GenBank	
*Apodemus agrarius*	34	12	2	2	MuApAg-11	SFMd2–1	MN266871	219	100	96	helix-turn-helix transcriptional regulator	*E. coli* O145:NM strain FWSEC0002 (CP031919.1)	Pathogenic *E. coli*
					MuApAg-13	SFMW-16	MN266867	456	100	100	*stxA2* gene (Shiga toxin Stx2 subunit A)	*E. coli* O157:H7 strain ECP17–1298 (CP040570.1)	
*A. peninsulae*	6	0	–	–	–	–	–	–	–	–		–	
*A. sylvaticus*	3	3	1	1	MuApSy-3	MWM3_9	MN266868	461	100	100	*stx2gA* (shiga toxin 2 g subunit A)	*E. coli* O157:H7 strain COPRO21317 (CP035706.1)	
*Micromys minutus*	3	0	–	–	–	**–**	**–**	**–**	**–**	**–**	**–**	**–**	
*Myodes regulus*	3	1	0	0	CrMyRe-3	**×**	**–**	**–**	**–**	**–**	**–**	**–**	
*R. norvegicus*	3	2	2	2	MuRaNo-1	NRW1–14	MN266869	321	100	95	helix-turn-helix transcriptional regulator	*E. coli* strain 89–3156 (CP027366.1)	
					MuRaNo-3	NRW3–15	MN266870	327	99	95	helix-turn-helix transcriptional regulator	*E. coli* O145:NM strain FWSEC0002 (CP031919.1)	
Total (N)	52	18	5	5	**–**	**–**	**–**	**–**	**–**	**–**	**–**	**–**	

' × '= not detected and '-'= not tested.

**Table 5:** Prevalence of *E. coli* and Shiga toxin-producing *E. coli* in the six rodent species in South Korea.

		Culture detection		Molecular detection		

					% (positive individuals/ tested individuals) with Shiga toxin gene of *E. coli*	

Species	No. of individuals	% (positive individuals/ tested individuals) with *E. coli* EMB	% (positive individuals/ tested individuals) with Shiga toxin producing *E. coli* CT-SMAC	% (positive individuals/ tested individuals) with *E. coli* 16S rRNA gene	Shiga toxin gene (*stx1*)	Shiga toxin gene (*stx2*)
*Apodemus agrarius*	34	55.88 (19/34)	35.29 (12/34)	55.88 (19/34)	0 (0/34)	2.94 (1/34)
*A. peninsulae*	6	16.66 (1/6)	0 (0/0)	16.66 (1/6)	0 (0/6)	0 (0/0)
*A. sylvaticus*	3	100 (3/3)	100 (3/3)	100 (3/3)	0 (0/3)	33.33 (1/3)
*Micromys minutus*	3	0 (0/0)	0 (0/0)	0 (0/0)	0 (0/0)	0 (0/0)
*Myodes regulus*	3	33.33 (1/3)	33.33 (1/3)	33.33 (1/3)	0 (0/3)	0 (0/0)
*R. norvegicus*	3	66.66 (2/3)	66.66 (2/3)	66.66 (2/3)	0 (0/3)	0 (0/0)
Total (N)	52	50 (26/52)	34.62 (18/52)	50 (26/52)	0 (0/52)	3.84 (2/52)

## 2 Experimental Design, Materials, and Methods

### 2.1 Sample collection

We captured wild rodents using Sherman traps in various agroecosystems across South Korea (Table 1). Each captured wild rodent was placed into a disposable vinyl zipper bag and then released after collecting its feces. The fecal samples were brought to the laboratory in ice boxes and processed within three hours. The sample collections were conducted under the permission and guideline of local governments.

### 2.2 Detection of diarrheagenic *E. coli* bacteria

The *E. coli* and Shiga toxin-producing *E. coli*, bacteria in the fecal samples were detected by culturing them using the method described in a previous study [2]. The fecal samples (0.1 g to 1 g) were first cultured in 10 mL non-selective buffered peptone water (BPW) at 37 °C overnight and then a 10 µl enrichment broth of containing the samples was streaked with a loop onto the *E. coli*-selective eosin methylene blue Agar (EMB) media and Shiga toxin-producing *E. coli*-selective cefixime tellurite sorbitol MacConkey agar (CT-SMAC) media and incubated at 37 °C for 24-48 hrs. The plates were examined for colony forming units (CFU) and sub-cultivated was conducted on EMB so that pure colonies could be collected. The *E. coli* or Shiga toxin-producing *E. coli* colonies were confirmed based on their colony morphology [5]. *E. coli* (NCPP: 14,034) and *E. coli* O157:H7 (ATTC-95,150) were used as a positive control. The *E. coli* colonies produced metallic sheen color produced on EMB agar media. Sorbitol negative colonies (colorless) were detected onto CT-SMAC agar. From each plate, 2 to 3 colonies were picked from CT-SMAC media. We select colorless colony as *E. coli* O157:H7 positive colonies seem to be STEC-positive on culture media. The STEC colonies have morphological differences in CT-SMAC compared to the general *E. coli*. Finally, the STEC *E. coli* was detected based on morphology, PCR band, and sequence analysis.

### 2.3 Extraction of total genomic DNA and PCR amplification

The bacterial colonies on the selective media or differential media had been identified by their morphology and re-identified by PCR using molecular markers. The colonies were streaked onto nutrient agar media and then a single colony was collected using sterilized toothpicks. The colonies were incubated at 35 °C for 18 hrs in 5 ml lactose broth (LB) solution. Genomic DNA was extracted from 1 ml LB culture fluid using a DNeasy Blood and Tissue Kit™ according to the manufacturer's instructions (Valencia, CA, USA).

The PCR amplification was performed using a final 25 μL reaction volume containing 10 mMTris–HCl (pH 8.4), 50 mMKCl, 4 mM MgCl_2_, 200 mM of each dNTP, 50 pmol of each primer, 2 U ExTaq polymerase, and 1 μL of genomic DNA. The *E. coli* colonies on the EMB were molecularly identified by PCR amplification of the bacterial 16S rRNA gene which was performed using the HVR primer set [6]. The Shiga toxin-producing *E. coli* colonies on CT-SMAC were molecularly identified by PCR amplification using the Stx1 and Stx2 primer set [7,8].

The PCR reaction was conducted using the following reaction conditions: an initial denaturation for 5 min at 94 °C, followed by 35 cycles of denaturation for 1 min at 94 °C, an annealing temperature of 55 °C (HVR, Stx1, and Stx2 primers) for 60 sec, extension for 1 min at 72 °C, and then a final extension for 10 min at 72 °C. The PCR products were subjected to electrophoresis in 1.0% agarose gel and purified using a DNA gel extraction kit (Qiagen, Valencia. CA, USA). The purified PCR products were sent to Macrogen (South Korea) for sequencing. The obtained sequences were compared with other homologous sequences deposited in GenBank using BLASTN2.2.31+ [1].

## Ethics Statement

The sample collections were conducted under the permission and guideline of local governments.

## Declaration of Competing Interest

The authors declare that they have no competing interest or financial relationships which have influenced the work reported in this article.

## Appendix Supplementary materials

Image, application 1
