# Supplementary material for: Prevalence data of diarrheagenic E. coli in the fecal pellets of wild rodents using culture methods and PCR assay
Source: Data Brief. 2020 Oct 22;33:106439. doi: 10.1016/j.dib.2020.106439 (PMC7609732; doi:10.1016/j.dib.2020.106439)
Supplement: Supplementary file 1 [file mmc1.docx]

**Supplementary data (Fig S1-S4).**

**-**

**22**

**12**


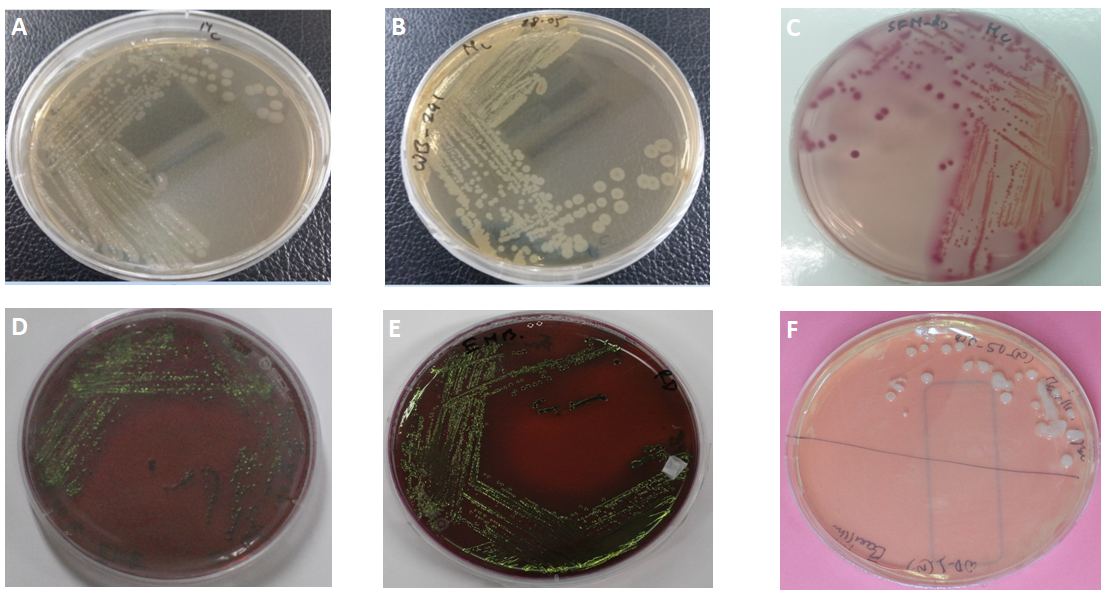


**Fig.S1**. **A.** Sorbitol negative colonies (white color or off white color) were observed from *Rattus norvegicus* fecal culture on cefixime tellurite sorbitol MacConkey agar (CT-SMAC) agar. **B.** Reference *Escherichia coli* O157:H7 (ATTC-95150) strain produced sorbitol negative colonies (white color or off white color) from (CT-SMAC) agar. **C.** Sorbitol positive colonies (pink or red color) were observed from *R. norvegicus* fecal culture on CT-SMAC agar. **D.** *Escherichia coli* positive colonies (metallic sheen color) were produced from *Apodemus agrarius* fecal culture on Eosin Methylene Blue Agar (EMB). **E.** Reference *E. coli* NCCP-14034 was observed positive colonies (metallic sheen color) on EMB agar media. **F.** *E. coli* grows on TSA agar media from *R. norvegicus* fecal.

**11**

**10**

**9**

**8**

**7**

**6**

**5**

**1**

**2**

**3**

**4**

**22**

**M**

**26**

**25**

**24**

**23**

**+**

**-**

**22**

**21**

**20**

**12**

**14**

**19**

**18**

**17**

**16**

**15**

**13**

**M**


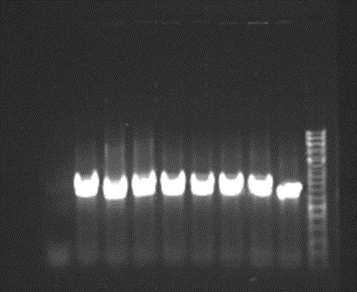

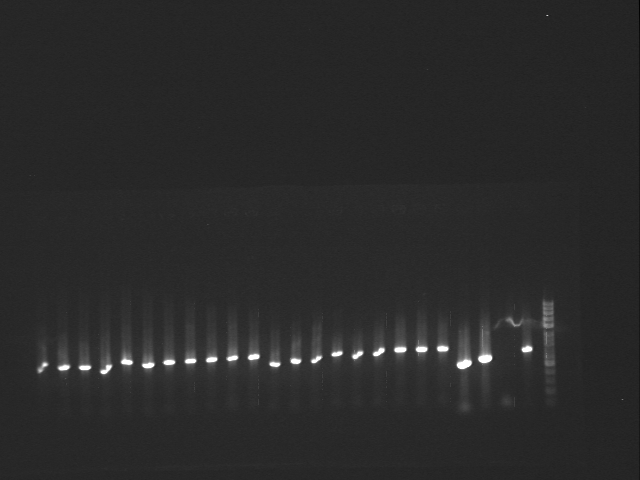


**1000**

**1000**

**500**

**500**

**300**

**400**

**100**

**200**

**400**

**300**

**200**

**100**

**Fig S2**. **PCR amplification of Bacterial 16S rRNA genes with HVR (V1S-F and V3A-R) primer set.** A single colony was randomly selected from each of 26 EMB agar plates with *E. coli* positive colonies. PCR bands were shown in all of the 26 single colonies selected from 26 EMB agar plates. PCR bands ‘M’ indicate DNA 100bp marker. Genomic DNA of *Escherichia coli* (NCPP: 14034) used as positive control (+) and only PCR mixture without genomic DNA of samples was used as negative control (-). Target band of amplified HVR primer is 683bp. Each sample is indicated by host individual ID with bacterial colony ID in parenthesis. The gel lane numbers are as follows: Lane No.1=MuApAg-7 (M1-6); No.2=MuApAg-10 (SFM-d-1); No.3=MuApAg-11 (SFM-d-2); No.4=MuApAg-12 (SFM-d-3); No.5=MuApAg-13 (SFMW); No.6= MuApAg-14 (SFM_1_BKNP); No.7=MuApAg-15 (SFM_2_BKNP); No.8=MuApAg-16 (SFM_3_BKNP); No.9=MuApAg-17 (SFM_4_BKNP); No.10=MuApAg-18 (M_1_Sobeksan_edit); No.11=MuApAg-19 (M_2_S); No.12=MuApAg-20 (M_3_Sobeksan_edit); No.13=MuApAg-22 (M_4_S); No.14= MuApAg-23(M_6_S); No.15= MuApAg-24(M_7_S); No.16= MuApAg-25(M_8_S); No.17=MuApAg-26(M_9_S); No.18= MuApAg-27(M_10_S); No.19= MuApAg-28(M_11_S); No.20= MuApPe-1 (M2M-3); No.21= MuApSy-1 (MWM-1); No.22= MuApSy-2 (MWM2-iii); No.22= MuApSy-2 (MWM2-iii); No.23= MuApSy-3 (MWM3); No.24= CrMyRe-3 (SFM); No.25= NuRaNo-1 (SFM_3_BKNP); No.26= NuRaNo-3 (SFM_4_BKNP). Target band of amplified HVR primer is 683bp. More details of sample information are provided in Table 1.


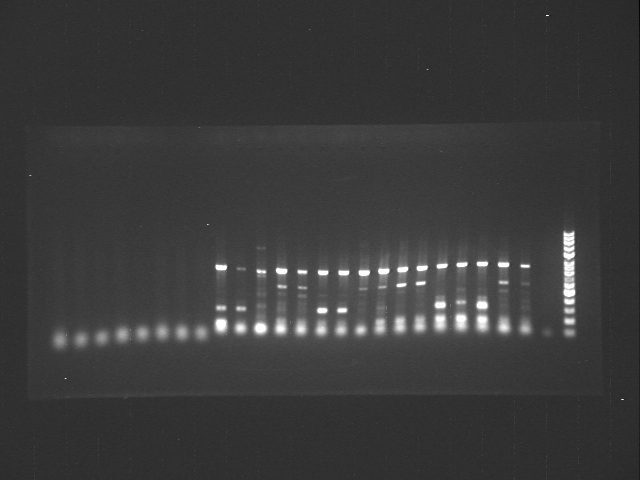


**1**

**2**

**3**

**4**

**5**

**6**

**7**

**8**

**9**

**10**

**11**

**13**

**15**

**14**

**12**

**+**

**-**

**100**

**200**

**400**

**1000**

**300**

**500**

**M**


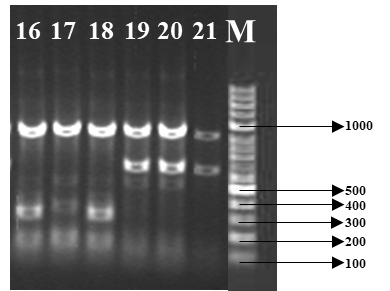


**Fig S3.** **PCR amplification of Shiga toxin genes with Stx1 primer set.** One or more pure colonies were randomly selected from all of 18 CT-SMAC positive agar plates with Shiga toxin-producing *E. coli* colonies: two single colonies (Lane No. 5 and 6) from MuRaNo-1 and three single colonies (Lane No. 15, 16 and 17) from MuApSy-3, and a single colony from each of the others. PCR bands were shown in all of total 21 single colonies selected from 18 CT-SMAC positive agar media. Genomic DNA of *Escherichia coli* 0157:H7 (NCPP: 15739) used as positive control (+) and only PCR mixture without genomic DNA of samples was used as negative control (-). PCR band ‘M’ indicate DNA 100bp marker. Target band of amplified *stx1* gene is 894bp. Each sample is indicated by host individual ID with bacterial colony ID in parenthesis. The gel lane numbers are as follows: Lane No.1=MuApAg-18 (M1_S); No.2= MuApAg-19 (M2_S); No.3=MuApSy-1 (MWM1_10); No.4=MuApSy-2 ((MWM2_4); No.5= MuRaNo-1 (NRW1-14); No.6= MuRaNo-1 (NRW1_LB); No.7= MuRaNo-3 (NRW3-15); No.8= MuApAg-11 (SFMd2-1); No.9= MuApAg-12 (SFMd3-24); No.10= MuApAg-7 (SFM1_2); No.11= MuApAg-13 (SFMW-16); No.12= MuApAg-23 (M6_S); No.13= MuApAg-28 (M11_S); No.14= CrMyRe-3 (MR-294); No.15=MuApSy-3 (MWM2_5); No.16=MuApSy-3 (MWM3_7); No.17=MuApSy-3 (MWM3_9); No.18=MuApAg-14 (SFM1_BKNP); No.19=MuApAg-15 (SFM2_BKNP; No.20=MuApAg-16 (SFM3_BKNP); No.21=MuApAg-17 (SFM1_BKNP). In detail sample and sequence information is provided in Table 1&3.


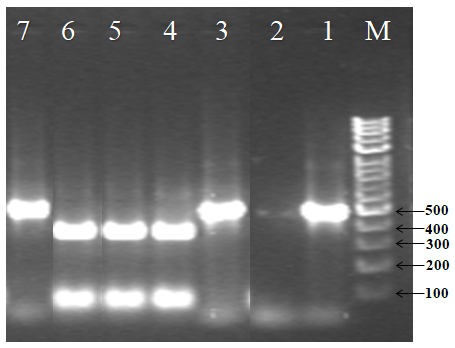


**Fig S4.** PCR **amplification of Shiga toxin genes with the Stx2 primer set.** A single colony was randomly selected from each of CT-SMAC positive agar plates with white color *E. coli* 0157:H7 positive colonies. Genomic DNA of *Escherichia coli* 0157:H7 (ATCC-95150) used as a positive control (+) and only PCR mixture without genomic DNA of samples was used as a negative control (-). PCR band ‘M’ indicate DNA 100bp marker. The target band of amplified *stx2* gene is 482bp. Each sample is indicated by host individual ID with bacterial colony ID in parenthesis The gel lane numbers are as follows: Lane No.1=MuApAg-11 (SFMd2-1); No.2= MuApAg-13 (SFMW-16); No.3=MuApSy-3 (MWM3_9); No.4=MuRaNo-1(NRW1-14); No.5= MuRaNo-3 (NRW3-15). In detail sample and sequence information had been provided in Table 1&4.
